# Sensori-Motor Learning with Movement Sonification: Perspectives from Recent Interdisciplinary Studies

**DOI:** 10.3389/fnins.2016.00385

**Published:** 2016-08-25

**Authors:** Frédéric Bevilacqua, Eric O. Boyer, Jules Françoise, Olivier Houix, Patrick Susini, Agnès Roby-Brami, Sylvain Hanneton

**Affiliations:** ^1^STMS Ircam-Centre National de la Recherche Scientifique-UPMCParis, France; ^2^UMR7222 ISIR - Université Pierre et Marie CurieParis, France; ^3^UMR 8242 Centre National de la Recherche Scientifique - Université Paris DescartesParis, France

**Keywords:** sonification, movement, learning, sensori-motor, sound design, interactive systems

## Abstract

This article reports on an interdisciplinary research project on movement sonification for sensori-motor learning. First, we describe different research fields which have contributed to movement sonification, from music technology including gesture-controlled sound synthesis, sonic interaction design, to research on sensori-motor learning with auditory-feedback. In particular, we propose to distinguish between *sound-oriented* tasks and *movement-oriented* tasks in experiments involving interactive sound feedback. We describe several research questions and recently published results on movement control, learning and perception. In particular, we studied the effect of the auditory feedback on movements considering several cases: from experiments on pointing and visuo-motor tracking to more complex tasks where interactive sound feedback can guide movements, or cases of sensory substitution where the auditory feedback can inform on object shapes. We also developed specific methodologies and technologies for designing the sonic feedback and movement sonification. We conclude with a discussion on key future research challenges in sensori-motor learning with movement sonification. We also point out toward promising applications such as rehabilitation, sport training or product design.

## 1. Introduction

The idea of using auditory feedback in interactive systems has recently gained momentum in different research fields. In applications such as movement rehabilitation, sport training or product design, the use of auditory feedback can complement visual feedback. It reacts faster than the visual system and can continuously be delivered without constraining the movements. In particular, movement sonification systems appear promising for sensori-motor learning in providing users with auditory feedback of their own movements. Generally, sonification is defined as the use of non-speech audio to convey information (Kramer et al., 1999). Nevertheless, research on movement sonification for sensori-motor learning has been scattered in totally different research fields. On the one hand, most neuroscience and medical experiments have made use of very basic interactive systems, with little concern for sound design and the possible types of sonification. On the other hand, novel sound/music interactive technologies have been developed toward artistic practices, gaming or sound design, with little concern for sensori-motor learning.

Clearly, there has been a lack of overlap between all these different disciplines, which would each benefit from more exchanges on tools, methods and knowledge. This rationale motivated us to initiate an interdisciplinary project that focused on sensorimotor learning in movement-based sound interactive systems^1^. Overall, this body of work, that we partially present in this *Perspective* paper, allowed us to establish general principles on movement sonification and to formalize fundamental questions that should be addressed in future research.

The paper is structured as follows. First, we recall related works in interactive music systems, human-computer design, sonic interaction design, and movement sonification for sport and rehabilitation. Second, we report on the questions and results we obtained in our project. Third, we discuss key research questions that should open a broad discussion.

## 2. Intersecting research on sound, movement, and interaction

Different types of interactive systems can produce sound based on human movement. Movement parameters are typically obtained from motion capture systems—such as optical motion capture, cameras, or inertial measurement units—and the sound can be rendered continuously using various types of real-time sound synthesis methods. In this paper, we restrain the discussion to interactive systems built with a deterministic *mapping* between movement and sound parameters (Dubus and Bresin, 2013). As described in the next section, these technologies have been developed in different contexts focusing either on sound or on movement aspects.

### 2.1. Movement-based interfaces for sound production and expression

The music technology research community has long been concerned with gestural and bodily control of sound^2^. Technologies for movement capture, analysis, recognition and interaction design have been developed and reported in the *sound and music computing* literature. In particular, the so-called *mapping* between movement parameters and sound synthesis parameters has been formalized and categorized (Hunt et al., 2003; Wanderley and Depalle, 2004). Methods and tools have been developed and are available for research communities (Leman, 2008; Schnell et al., 2009; Fiebrink and Cook, 2010; Bevilacqua et al., 2011). Surprisingly, though, sensori-motor learning has been rarely studied explicitly in such electronic or digital musical instruments.

In musical applications, the goal of the interaction is often to produce a specific *sound*. Therefore, we propose to refer to such tasks as *sound-oriented tasks*, during which the focus of the user's attention is drawn toward the sound produced by the interactive system. In general, the users must adapt their movement to the interface and gain expertise to achieve high control of sound production and musical expressivity. We explicitly used the concept of *sound-oriented task* to demonstrate how auditory feedback can be used in sensori-motor adaptation (Boyer et al., 2014). This important point will be further discussed in Section 3.

### 2.2. Movement sonification for sensori-motor learning

On the other side of the spectrum lie works on sensori-motor learning *per se*. The large majority of neuroscience papers on the human motor system only deals with visual, haptic and vestibular sensory inputs and rarely mentions the auditory modality. Historically, most papers reporting on auditory-motor mechanisms are concerned speech learning and production. Due to promising applications in movement learning, mostly in sport and rehabilitation, there has recently been an increasing number of studies showing the potential interest of auditory feedback (see Sigrist et al., 2013 for a review). Nevertheless, the technology used in these studies remains generally rudimentary, considering only simple movement-to-sound mapping using parameters such as audio energy and pitch (Dubus and Bresin, 2013).

Generally, the tasks described in such research correspond to what we call *movement-oriented tasks*, where the attention (and the instruction) is put on the movement itself. Movements are thus designed to exhibit specific characteristics (e.g., exercises in rehabilitation) or fully constrained by the application (e.g., specific movements that must be mastered in a given sport). The auditory exteroceptive concurrent feedback either informs whether the movement is properly executed (KR: Knowledge of Results) or how it is executed (KP: Knowledge of Performance) (Schmidt, 1988; Cirstea et al., 2006).

It is worth noting that the beneficial effect of music therapy for sensori-motor rehabilitation is now well recognized, particularly in stroke patients (Ripollés et al., 2015) and in other neurological diseases such as Parkinson (Thaut, 2015) where the synchronization of rhythmic auditory cues is proven to improve gait and motor activity (Schiavio and Altenmüller, 2015). The effect of music training is probably not only due to motivation and psychosocial factors linked with community practicing but also to the multisensory feedback linked to musical motor actions and the brain plasticity it induces (Schlaug, 2015). Rhythmic cues are an important support during music execution (Schneider et al., 2007). Less is known about the effect of continuous sound or music feedback on discrete movements of the upper-limb. Recent evidence suggests that such tasks performed with continuous sound feedback could improve the performance and facilitate learning (Rosati et al., 2012). Thus, sonification has been proposed during rehabilitation, in isolation or to augment other exercise based methods (Scholz et al., 2014).

## 3. Research questions and fundamental studies

**Figure 1:**
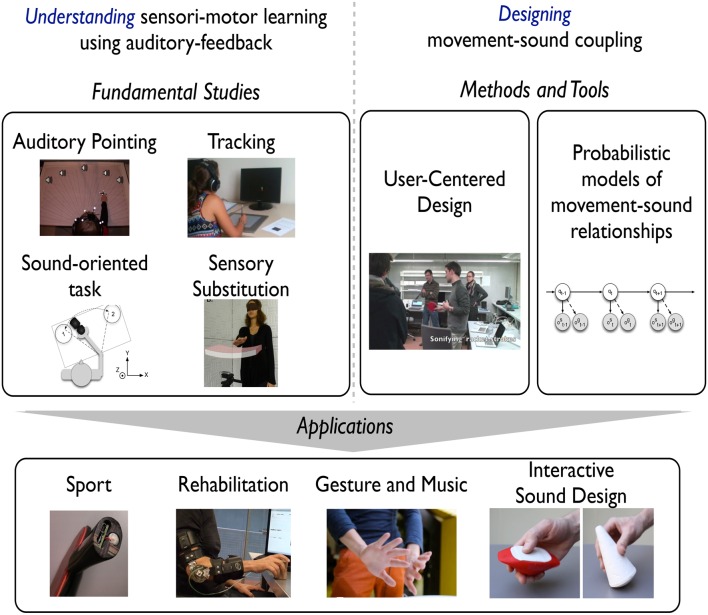
**This figure summarizes the interdisciplinary research we conducted in the ***Legos*** project, from fundamental research, methods and tools, to applications**.

We report below the different research questions we have investigated (see Figure 1), covering fundamentals studies, methods and tool development. In particular, we describe in this section the fundamental and methodological aspects of our experimental studies on the influence of continuous and concurrent auditory feedbacks.

### 3.1. Can auditory feedback modify and/or improve movement performance?

We investigated movement sonification in a visuo-motor tracking task (Boyer, 2015). In this case, we compared the sonification of three different variables: the visual target presented on a screen, the participant's pointer (i.e., movement hand) and the online error between the target and the pointer. In the three conditions, we found a positive effect of the auditory feedback for improving the tracking accuracy. Interestingly, the sonification of the hand movement seems in this case to favor an increase of the average movement energy, even after a long exposure to the task, and to improve retention.

Another study focused on a pointing task to an unseen spatialized auditory target, in which we evaluated the role of the target sound duration and the movement sonification (Boyer et al., 2013a). A long duration target presentation improved the pointing accuracy, highlighting the contribution of neuronal integration processes of the auditory information. The hand movement sonification was not found useful in this case, which might be explained by the complexity of the perception of two different spatialized sound sources (target and sonified hand).

Tajadura-Jiménez et al. (2014) also showed that in a touch task, interactive auditory feedback could modify the user's behavior, precisely the hand velocity and finger pressure. Finally, we found that movement sonification could be used to stabilize the performance of newly learned gestures (Françoise et al., 2016).

### 3.2. Can the presentation of a specific sound serve to specify a movement?

We investigated whether auditory feedback can be designed for guiding users in the performance of a specific movement. For example, we built an interactive system where participants had to discover how to move an object on a table using solely the auditory feedback (Boyer et al., 2014). They were asked to pay attention to specific sound features, which corresponds to what we define as a *sound-oriented task*. The whole movement was continuously sonified with sound properties depending on the error between the performed and targeted velocity profiles. Globally, we found that such an auditory feedback was effective to guide participants to learn to perform a predefined velocity profile. Also, after a first stage of exposure with a fixed velocity profile, movement adaptation was also observed when modifying the target profile (without informing the participants). This confirmed similar results obtained by Rath and Rocchesso (2005) and Rath and Schleicher (2008).

Importantly, a large variability was found between participants, which could be partially explained by the fact that such a task (i.e., performing a specific movement being guided by sound feedback) was totally unfamiliar to the participants. It is also likely that each subject exhibits different audio-motor ability.

### 3.3. Sensory substitution: can sound replace another modality?

We explored a case of sensory substitution where participants had to estimate the curvature of a virtual shape from auditory feedback (Boyer et al., 2015; Hanneton et al., 2015). In the experiment, users received continuous auditory feedback when ‘touching’ the virtual surface. While the accuracy of participants' estimation of the curvature was inferior to published results with tangible surfaces, we found that the auditory feedback can be effective for such a task, especially when the sound responds to the hand velocity. Most interestingly, different strategies on the use of the movement-sound interaction were observed between users: some persons tend to gently tap perpendicularly to the surface, while others prefer to explore the surface with large lateral movements. This also here indicates large discrepancies between participants in transferring movement sonification information.

### 3.4. Can (interactive) sound alter perception and emotion?

As we just reported, people can use the auditory channel to adapt their movements. Nevertheless, little is known about the subjective changes (for the users' perception) of the sound and the movement, as well as possible change in their emotional state. In a tapping task with an artificial auditory feedback, the emotional response has been found to be affected by the congruence between the sound energy and the tapping dynamics (Tajadura-Jimenez et al., 2015). In particular, audio-motor incongruences can lead to unpleasant experiences, which shows that expectation of the user for the audio feedback might be crucial for integrating the feedback. The artificial sound feedback of touch can also alter the texture perception, such as the coldness or material type (Tajadura-Jiménez et al., 2014).

Beyond fundamental neuroscience research, such investigations—that confirm other studies on multimodal sensory integration (Zampini and Spence, 2004; Maes et al., 2014),—have high impact potential applications for diminishing pain (Singh et al., 2016) or effort perception (Fritz et al., 2013).

## 4. Designing movement-sound interaction

The various results we gather indicates that the effect of the sonification might depend on specific aspects of the interaction design, which confirmed previous studies. In particular, the sound, and more specifically the congruence between the movement and sound, can strongly modify the user experience and therefore the effectiveness of the feedback. In Castiello et al. (2010), it was shown that the effect on the action of reaching and grasping an object is favored (in terms of movement duration) for congruent conditions, when the sound corresponds to the material covering the object to grasp, compared to incongruent conditions. In Susini et al. (2012), congruent sound-action conditions in terms of naturalness were found to be determinant in the appraisal of the sounds of everyday objects. These findings call for improving methodologies for the design of such sound interactive systems.

Building upon previous results (Rocchesso et al., 2009; Franinović and Serafin, 2013), we developed user-centered methodologies based on participatory design workshops. A central idea was to explore strategies combining the analysis of various objects' affordances with established sound and action taxonomies (Houix et al., 2014, 2015). The design of the movement-sound can be leveraged by taking advantages of users expectancy on the auditory feedback. In such a case, we refer to *ecological* relationships between action and sound.

The notion of object affordances can also be extended to sound, by questioning reversely which movement could be performed to match a given sound (Caramiaux et al., 2014a,b). Following such premises, we developed a method called *mapping by demonstration*, that allows to program an interactive systems based on movement performed while listening to a sound (Françoise, 2015). Such an approach can leverage known association between movement and sound feedback, and is particularly adapted for user-centered methodology in the design of interactive systems.

## 5. Discussion and future research challenges

We discuss here some of the research questions we mentioned in the previous sections, and propose new steps that we think as central for future research.

First, auditory feedback can be designed to convey different type of information. A first approach is to inform continuously on the error between the performed movement and a “normal” movement. In this case, the learning or adaptation is explicit. The alternative approach is to provide users with a real-time movement sonification independently of a reference to a “normal movement.” In this case, implicit learning is in play. The comparison between these two approaches remains to be carefully investigated, both in term or learning speed and retention. Our results (Boyer et al., 2014; Boyer, 2015) show that these two approaches are in fact complementary and the combination of both can be beneficial. Nevertheless, more studies are necessary to clarify the different neural mechanisms that are implied:

- *Are the neural mechanisms for error-related auditory feedback different to those when movement sonification is used as a feedback on users' own movements?* In particular, can the movement sonification be considered as “enhancing proprioception” in integrating congruent information?
- *What is the role of the guidance hypothesis in these cases?* Can the constant use of the auditory feedback be detrimental for learning (i.e., the effect disappears when the feedback is removed). Such a point needs to be clarified for both approaches and has still insufficiently been studied.
- *Is the standard distinction between KR (knowledge of results) or KP (knowledge of performance) relevant when considering movement sonification?* Since both feedback types can occur simultaneously during real-time movement sonification (Boyer et al., 2014), specific formalization of auditory feedback types should be developed.

Second, the role of the sound characteristics remains elusive for quantifying the learning efficiency or learning rate. Reported results have been sometimes contradictory, and very different mapping or sound types have been equally successful. The role of the mapping or sound quality must be further studied, and we particularly propose to focus on two important questions:

- *How does a particular mapping can favor agency in the interaction?* The auditory feedback should be clearly predictable by the user. This requires to study both the sound and movement perception in an interactive context (Effenberg, 2005).
- *When is the auditory feedback perceived as motivational?* Fundamental studies of sensori-motor learning with auditory feedback has generally avoided to take into account possible emotional response to the auditory feedback. Nevertheless, even the simplest ones using pure tone might trigger positive or negative effects, which can potentially affect the task perception, adaptation and learning (Lemaitre et al., 2012).

Third, our studies as well as many other published results point toward a large variability between participants. Such findings might be put in parallel with the large variability found in rhythmic ability, which motivated the establishment of a standard test called BAASTA (Farrugia et al., 2012). We believe that such a test would be highly useful for movement sonification in interactive systems. This would represent a first step toward more reproducible results and build understanding of possible causes of this variability. This point is also crucial to develop real-world applications. Moreover, sound design applications provide extremely fruitful cases to study sound perception as an active and contextual process. In that new framework, sound perception studies should be redesigned in relation to gesture and to user's objectives.

As already mentioned, movement sonification or more generally the use of auditory feedback have been already proposed for specific applications. Beyond artistic communities which have already largely included movement-based interactive systems, the most prominent ones are rehabilitation (Robertson et al., 2009; Roby-Brami et al., 2014; Scholz et al., 2014; Katan et al., 2015), sport learning and training (Effenberg, 2004; Eriksson and Bresin, 2010; Boyer et al., 2013b) while human-computer interaction also shows a growing interests (Franinović and Serafin, 2013; Oh et al., 2013).

In stroke patients, the sound based therapies are specifically promising to target the impairment of the upper-limb. The contemporary guidelines for rehabilitation insist on the similarity between sensori-motor learning and recovery phenomenon. Thus, therapy should be improved both in quantity and quality: on the one hand it should be based on massive exercise repetitions, emphasizing on sensory-motor reciprocity and multisensory integration. On the other hand, the therapy should be adapted to the needs of each individual: the exercices should be shaped according to the precise capabilities of the person and should evolve according to his/her abilities and progress during learning. Sound feedback is frequently integrated into virtual and augmented reality rehabilitation training but its use is often limited to rhythmic auditory cues or reinforcement feedback signaling only the success to an exercise. We propose that sonification could be further developed to target specific impairments in stroke patients as a continuous feedback during movement execution. Sonification is particularly interesting to signal to the patients some impairment, which they might not be aware of, particularly if they have somatosensory impairments, for example error in direction, in coordination or lack of movement smoothness (Maulucci and Eckhouse, 2001), in coordination of reaching and grasping for prehension or in grasping to lift coordination (Hsu et al., 2012). Thanks to a braked elbow orthosis, we simulated the disrupted shoulder-elbow coordination observed in hemiparetic stroke patients and used this device to test sonification strategies that we developed to target shoulder elbow coordination. Further studies are needed in order to find a compromise between two possibly contradictory requirements: target the specific impairments of stroke patients and develop motivation linked to exploration of sophisticated auditory-motor coupling.

Beside the fundamental aspects we described about the understanding of the different auditory feedback mechanisms that can contribute to sensori-motor learning, the development of rigorous—and shared—sound design methodologies is crucial for grounding these applications. As a matter of fact, the use of sound in any technological applications could lead to user annoyance or discomfort, even if globally beneficial for movement training. We therefore advocate for more interdisciplinary research bringing together sound designer, musicians, engineers, cognitive scientists, to work toward efficient applications using movement-based sonification. One the one hand, the collaboration with sound artists and musicians is generally necessary to design pleasant and motivational interactive sound and music systems, on the other hand sound design research should further develop methods to assess naturalness and pleasantness of sonic interactive system (Susini et al., 2012).

## Author contributions

FB drafted the paper, and all the other authors (EB, JF, OH, PS, AR, SH) revised the article critically for important intellectual content. All authors contributed to the research project described in this Perspective article (i.e., Legos project) and participated in formalizing the research questions and perspectives. All authors gave their final approval for publication of this paper.

### Conflict of interest statement

The authors declare that the research was conducted in the absence of any commercial or financial relationships that could be construed as a potential conflict of interest.

## Footnotes

^1^Legos project, see http://legos.ircam.fr.

^2^See for example the community related to the NIME conferences (New Interfaces for Musical Expression) (Bevilacqua et al., 2013).
